# Development and Utilization of Functional Kompetitive Allele-Specific PCR Markers for Key Genes Underpinning Fiber Length and Strength in *Gossypium hirsutum* L.

**DOI:** 10.3389/fpls.2022.853827

**Published:** 2022-03-14

**Authors:** Lihua Li, Zhengwen Sun, Yan Zhang, Huifeng Ke, Jun Yang, Zhikun Li, Liqiang Wu, Guiyin Zhang, Xingfen Wang, Zhiying Ma

**Affiliations:** State Key Laboratory of North China Crop Improvement and Regulation, North China Key Laboratory for Crop Germplasm Resources of Education Ministry, Key Laboratory for Crop Germplasm Resources of Hebei, Hebei Agricultural University, Baoding, China

**Keywords:** functional markers, haplotype, kompetitive allele-specific PCR (KASP), marker-assisted selection (MAS), cotton fiber quality

## Abstract

Fiber length (FL) and fiber strength (FS) are the important indicators of fiber quality in cotton. Longer and stronger fibers are preferred for manufacturing finer yarns in the textile industry. Functional markers (FMs) designed from polymorphic sites within gene sequences attributing to phenotypic variation are highly efficient when used for marker-assisted selection (MAS) in breeding superior varieties with longer FL and higher FS. The aims of this study were to develop FMs *via* kompetitive allele-specific PCR (KASP) assays and to validate the efficacy of the FMs for allele discrimination and the potential value in practice application. We used four single-nucleotide polymorphism markers and 360 cotton accessions and found that two FMs, namely, D11_24030087 and A07_72204443, could effectively differentiate accessions of different genotypes with higher consistency to phenotype. The appeared frequencies of varieties harbored Hap2 (elite alleles G and T) with longer FL (> the mean of accessions with non-elite allele, 28.50 mm) and higher FS (> the mean of accessions with non-elite allele, 29.06 cN•tex^–1^) were 100 and 72.7%, respectively, which was higher than that of varieties harbored only on a single elite allele (G or T, 77.9 or 61.9%), suggesting a favorable haplotype for selecting varieties with superior FL and FS. These FMs could be valuable for the high-throughput selection of superior materials by providing genotypic information in cotton breeding programs.

## Introduction

Upland cotton (*Gossypium hirsutum* L.) holds a predominant position as a cash crop by supplying largely natural fibers for the global textile industry (Paterson et al., 2012). Fiber length (FL) and fiber strength (FS) are the important indicators of fiber quality in cotton (Paterson et al., 2003). Longer and stronger fibers are preferred for manufacturing finer yarns in the textile industry. Therefore, a large number of genes involved in fiber development have been identified in the past few years (Li et al., 2005; Shi et al., 2006; Qin and Zhu, 2011; Ma et al., 2018; Zhang et al., 2019). The gene *GhFL2* encoding Kip-related protein 6 (KRP6) was identified as a key gene for fiber elongation in our previous study (Ma et al., 2018). The molecular genetic basis of fiber elongation attributed to a non-synonymous mutation in the first exon (G altered to T) resulted in an amino acid change from Glu (E) to Asp (D). Overexpression of the gene with GG genotype increased the leaf trichomes 146.32 μm compared with wild type in *Arabidopsis* (Ma et al., 2018). Moreover, this gene was also detected by the combined analyses of eQTL and GWAS and was considered as playing a primary regulatory role of other genes responsible for FL (Li et al., 2020). The deletions in the 5′ untranslated region (UTR), intronic, and 3′ UTR of *GhFL2* were also identified to be significantly associated with FL in our recent study (Ma et al., 2021). Another important gene, *Gh_A07G1769*, encoding chaperone DnaJ-domain superfamily protein, was deemed as a causal gene underpinning the FS of cotton (Ma et al., 2018). Three polymorphic single-nucleotide polymorphisms (SNPs) (A07_72203768, A07_72204322, and A07_72204443) were identified in the coding sequence of the gene resulting in the amino acid change from Val (V) to Ile (I), Thr (T) to Ser (S), and Lys (K) to Glu (E), respectively. Therefore, these two genes were suggested as target genes for selecting superior varieties in breeding programs. However, the utilization of these genes in breeding selection is still not in practice.

In cotton, numerous genetic markers that contribute to detecting many important phenotypes for fiber quality (Islam et al., 2016; Fang et al., 2017; Keerio et al., 2018; Liu et al., 2018; Ma et al., 2018; Su et al., 2018), disease resistance (Shen et al., 2006; Mei et al., 2014; Palanga et al., 2017; Zhang et al., 2021), and early maturity (Stiller et al., 2004; Li et al., 2016) have been identified. The availability of these genetic markers will accelerate the development of molecular breeding. However, many of these genetic markers were somewhat distant from target genes and their predictive value was dependent on the degree of linkage to genes (Bagge et al., 2007), and it was also proved that the efficacy of marker-assisted selection (MAS) would be greatly diminished by the occasional uncoupling of the marker from the trait during the many cycles of meiosis in breeding programs when used the tightly linked markers, which would result in error selection for traits of interest (Perumalsamy et al., 2010). As a result, relatively few linked markers are used in cotton breeding.

In contrast, the utilization of functional markers (FMs) derived from polymorphic sites within gene sequences affecting phenotypic variation is considered to be more accurate (Bentley et al., 2014). FMs to assist selection could also be more efficient for accelerating the breeding process, which could result in quicker line development and variety release (Andersen and Lübberstedt, 2003; Zhou et al., 2013). Therefore, FMs based on SNPs are thought to be powerful for MAS in the breeding program. For instance, five functional SNP markers based on soybean genes, *FAD2-1A* and *FAD2-1B*, contributing to oleic acid levels, and *FAD3A* and *FAD3C*, contributing to linolenic acid levels (Shi et al., 2015), and a superior allele of TaSnRK2.10-A contributing significantly to wheat one-thousand-grain weight (Wang et al., 2014), were identified. In cotton, some potential functional SNP markers from expressed sequence tags (EST) (Li et al., 2014); three SNP markers for specific phytochrome genes, such as *PHYA1*, *PHYB*, and *HY5* (Kushanov et al., 2016); 10 SNP markers for cellulose synthase genes (Lin et al., 2012); and four SNP markers for male sterility genes were developed (Feng et al., 2015). All these FMs provided molecular tools for the selection of interesting traits. However, their application remains limited due to higher costs and lower throughput.

The kompetitive allele-specific PCR (KASP), a single-step genotyping technology, can type SNPs at specific sites relying on allele-specific oligo extension and fluorescence resonance (Semagn et al., 2014). It has the major advantages of low cost and high throughput for genotyping of SNPs (Neelam et al., 2013; He et al., 2014). The objectives of this study were (1) to develop functional KASP markers based on the mutations of important FL and FS genes, *GhFL2* and *Gh_A07G1769*; (2) to evaluate the efficacy of the markers for allele discrimination and accuracy; and (3) to assess the potential application value of the markers and screen out the more favorable haplotype for MAS.

## Materials and Methods

### Plant Materials and Generation of Phenotypic Data for Fiber Length and Fiber Strength

A total of 360 upland cotton (*G. hirsutum* L.) accessions were used in this study. These accessions were derived from various cotton-producing areas, including 146 from the Yellow River basin; 69 from the Yangtze River basin; 25 from the Northwest Inland, China; 55 from Cotton Research Institute, Chinese Academy of Agricultural Sciences (CRI, CAAS); 44 from abroad; and others from China with no detailed information (Supplementary Table 1). Field trials of the accessions panel were conducted in eight environments, including Baoding, Xinji, Hejian, Qingxian in Hebei Province, and Yacheng in Hainan Province in 2014 and Xinji, Qingxian, and Yacheng in 2015. All field trials were conducted in a randomized complete block with two replicates for each accession in 2014 and three replicates in 2015. The 20 samples of naturally open bolls were handpicked from the central part of the plant for each accession when matured. The fiber quality was measured using a high volume instrument at the Fiber Quality Supervision and Testing Center, the Ministry of Agriculture of China in Anyang, Henan Province. The phenotype and genotype of a core collection were used for haplotype validation of 419 cotton accessions from the previous study (Ma et al., 2018).

### Primer Design for Important Genes *GhFL2* and *Gh_A07G1769*

A total of four SNPs, D11_24030087 for FL and A07_72204322, A07_72203768, and A07_72204443 for FS, were used to develop FMs according to the SNP mutation sites located in the genes *GhFL2* and *Gh_A07G1769*. The KASP primers were developed following the standard guidelines. Flanking regions of different alleles at each locus were obtained from the DNA genomic sequence database of TM-1 (NAU-NBI version 1.1) (Zhang et al., 2015). We extracted 100 nucleotides on either side of each specified SNP site and then sent them to LGC Limited, United Kingdom, for primer design. The designed KASP assay mix comprised three primers: two forward primers designed from allele-specific primer, carrying the standard FAM and HEX fluorescence, and the reverse primer designed from the common primer of the corresponding sequence. The primer sequences are shown in Table 1.

**TABLE 1:** List of primer sequences used for KASP assays.

Marker	Allele	Primer (5′–3′)	Sequence
A07_72204443	C/T	Forward_FAM	GGGAACTAGTCTCATTAGAATCGAC
		Forward_HEX	GGGAACTAGTCTCATTAGAATCGAT
		Common reverse	TACTAGCCCTCCCCCATACATGAA
A07_72204322	G/C	Forward_FAM	ATTCCACTCTTTACACAACCTACTAC
		Forward_HEX	CCACTCTTTACACAACCTACTAG
		Common reverse	GTGGTCTTGAAGGAGGGGGTCTT
A07_72203768	T/C	Forward_FAM	GAGAGGGAAAGGGAGGAGAAGA
		Forward_HEX	AGAGGGAAAGGGAGGAGAAGG
		Common reverse	TCCCTCTCTTTTTCAAGGCTTCTTTGTT
D11_24030087	G/T	Forward_FAM	ATTTGGAGAAATCGCAGTCATGGAG
		Forward_HEX	AAATTTGGAGAAATCGCAGTCATGGAT
		Common reverse	CCGCCATGGCTCGTGTTCGAA

### Genomic DNA Extraction and Kompetitive Allele-Specific PCR Genotyping

The total genomic DNA of each accession was extracted from young leaves following a modified cetyltriethylammnonium bromide (CTAB) method (Zhang and Stewart, 2000). The quality and concentration of DNA were measured using NanoDrop 2000 UV spectrophotometer (Thermo Fisher Scientific, Waltham, MA), and working solutions were controlled at a concentration of 20–30 ng/μl.

The genotyping assays were performed on a 384-well plate. The reaction volume of 384-plate format PCR system can be established as 3.04 μl reactions (1.5 μl of template DNA, 1.5 μl of 1 × KASP mix, and 0.04 μl of primer mix). The amplifications were performed using a GenePro™ Thermal Cycler (Hydrocycler) with the following cycling conditions: 94°C for 15 min, 10 touchdown cycles (94°C for 20 s; touchdown at 61°C, dropping to –0.6°C per cycle 60 s) and then followed by 26 cycles of amplification (94°C for 20 s, 55°C for 60 s). After the run was completed, reaction plates were read and the data were analyzed using StepOne Software version 11.1 (LGC Limited, United Kingdom).

### Application of Composite Kompetitive Allele-Specific PCR Markers and Further Validation of Haplotype

The FL marker D11_24030087 (G/T) was used first to discriminate the genotypes of materials, and then the materials with favorable alleles were selected. Second, these selected materials were further genotyped using the FS marker A07_72204443 (C/T). Finally, the materials with both favorable alleles were screened out.

### Statistical Analyses

Statistical analyses for phenotypic traits were conducted using SPSS 20.0. Allelic effects for a single KASP marker were tested for statistical significance using a *t*-test. The effects of haplotypes on phenotypes were determined using a one-way analysis of variance (ANOVA). The significance level was set at *P* = 0.05 or 0.01. Traits were treated as dependent variables, and haplotypes served as independent variables.

## Results

### Phenotypic Characteristics of Fiber Length and Fiber Strength

**FIGURE 1:**
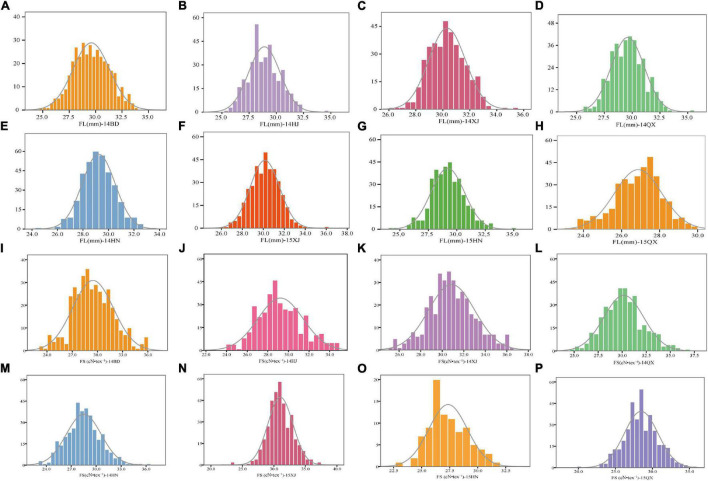
Phenotypic distribution of 360 upland cotton accessions for FL and FS under eight environments **(A–P)**. BD, Baoding; HJ, Hejian; XJ, Xinji; QX, Qingxian (in Hebei Province); HN, Yacheng (in Hainan Province) in 2014; XJ, Xinji; QX, Qingxian; HN, Yacheng in 2015.

A panel comprising 360 upland cotton accessions was grown over eight environments to obtain the phenotypic characteristics of FL and FS. All the accessions showed continuous distributions for FL and FS traits across eight various conditions (Figures 1A–P). The phenotypic data of FL in eight environments showed a wide range of variation from 22.22 to 36.14 mm and the FS ranged from 19.91 to 37.47 cN•tex^–1^, which showed abundant variations in the population (Table 2).

**TABLE 2:** Phenotypic variation for FL and FS traits of 360 accessions in eight environments.

Environment	Traits					
	FL (mm)			FS (cN•tex^–1^)		
	Max	Min	Mean	Max	Min	Mean
14-BD	33.58	24.66	29.21	35.80	23.30	29.41
14-HJ	34.78	25.60	28.78	34.80	24.20	29.18
14-XJ	35.20	25.14	30.14	36.30	25.20	30.81
14-QX	35.56	25.54	29.51	36.80	24.80	30.09
14-HN	33.60	24.50	29.23	33.30	23.30	28.47
15-XJ	36.14	25.63	30.05	37.47	23.23	31.02
15-QX	29.99	22.22	26.62	35.51	19.91	28.39
15-HN	34.80	24.15	29.03	31.61	22.70	26.62

### Development of Kompetitive Allele-Specific PCR Single-Nucleotide Polymorphism Marker for Gene *GhFL2*

**FIGURE 2:**
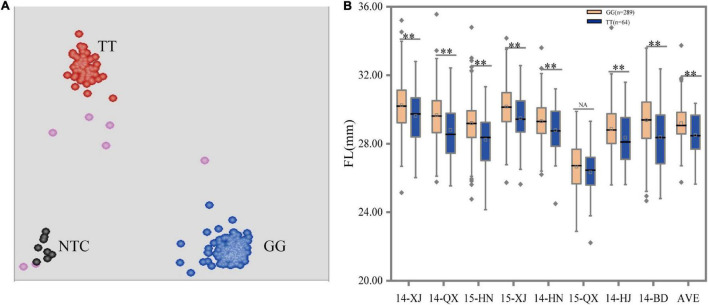
Scatterplots and box plots for marker D11_24030087 (G/T). **(A)** Clusters of accessions on the *X*- (FAM) and *Y*- (HEX) axes. The red, blue, pink, and black dots represent the homozygous, missing or failed data, and non-template control, respectively. **(B)** Allele effects were measured using different phenotypic data under eight environments. **, and NA indicate significant levels at *P* < 0.01 and no significant difference, respectively.

In our previous study, one SNP mutation, D11_24030087 (G/T), on the coding region of *GhFL2* was detected to be significantly associated with FL. This polymorphic site was located at the exon of *GhFL2* and was responsible for the variation in the FL trait (Ma et al., 2018). Therefore, it was identified as the genotyping target of the *GhFL2*. In this study, we designed an allele-specific primer for the variants of the alleles. A clear separation of two genotype clusters (GG and TT) with this marker was observed, no heterozygous genotype was discovered (Figure 2A). To validate the efficacy of the KASP markers for genotyping, we analyzed the 360 individuals in relation to genotype with phenotype. The group with the “GG” genotype included 289 lines with an average FL of 29.19 mm, which was significantly higher than the corresponding group with the “TT” genotype, including 64 lines with an average FL of 28.50 mm. The remaining accessions that failed to genotype were excluded from further analyses. Thus, the G-allele was considered as an elite allele for increasing FL. Furthermore, we also evaluated the efficacy of the marker using the phenotypic data under each environment and similar trends were detected except for 15-QX (Figure 2B, Table 3, and Supplementary Table 2). The above results demonstrated that this marker was effective for selecting accessions with longer FL in this validation population.

**TABLE 3:** Allelic effects of genes underpinning FL and FS traits in the population of 360 accessions.

Gene	Traits	Marker	Allele	AVE (mm)/(cN•tex^–1^)	Allele effect	Frequency%	*P*-value
						FL ≥ 28.50 FS ≥ 29.06	
*GhFL2*	FL	FL-24030087 (G/T)	G (289)	29.19	T < G 0.69	225 (77.9)	2.12E-06
			T (64)	28.50		31 (48.4)	
*Gh_A07G1769*	FS	FS-72204443 (C/T)	C (331)	29.06	C < T 1.09	159 (48.0)	0.0017
			T (21)	30.15		13 (61.9)	

*Significant differences are based on a probability (P) threshold of 0.01; direction of significance is shown by “<”; AVE, average phenotypic data under eight environments.*

### Development of Kompetitive Allele-Specific PCR Single-Nucleotide Polymorphism Marker for Fiber Strength Gene *Gh_A07G1769*

**FIGURE 3:**
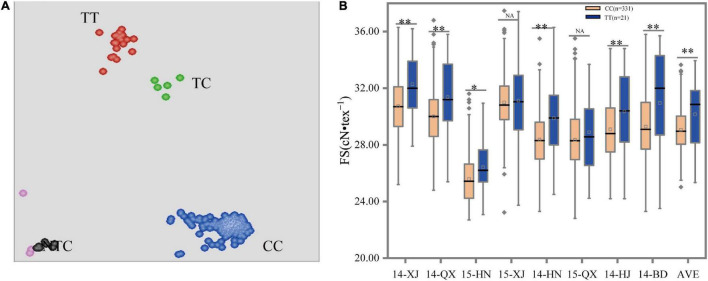
Scatterplots and box plots for marker A07_72204443 (C/T). **(A)** Clustering of accessions on the *X*- (FAM) and *Y*- (HEX) axes. The red, green, blue, pink, and black dots represent the homozygous, heterozygous, homozygous, missing or failed data, and non-template control, respectively. **(B)** Allele effects were measured using different phenotypic data under eight environments. *, **, and NA indicate significant levels at *P* < 0.05, *P* < 0.01, and no significant difference, respectively.

The mutations of three SNP markers, such as A07_72204322, A07_72203768, and A07_72204443, in the coding sequences of gene *Gh_A07G1769* resulting in amino acid change, were verified for deployed markers for the FS trait using the validation population. The result showed that only the marker A07_72204443 (C/T) could clearly distinguish type alleles CC, TC, and TT in the accessions (Figure 3A). The other two markers, namely, A07_72203768 and A07_72204322, generated only one genotype call, indicating that these markers had no diversity and were considered invalid for selecting individuals. The clusters amplified by the KASP marker of A07_72204443 (C/T) showed that the CC genotype, including 331 accessions with an average FS of 29.06 cN•tex^–1^, was significantly lower than the TT genotypes, including 21 lines with an average FS of 30.15 cN•tex^–1^. The heterozygous CT genotypes and accessions that failed to genotype were removed from subsequent analyses. To assess the utility of the developed KASP markers, the consistency between phenotype and genotype was also validated. The *t*-test showed that the marker had significant effects on FS based on the average phenotypic data under eight environments. Given the above results, the allele “T” was more superior to “C” for increasing FS (Figure 3B and Table 3). Additionally, we also validated the reliability of this marker separately using the phenotypic data under each environment. Results were consistent with the above results except for 15-XJ and 15-QX (Supplementary Table 3).

### The Application of Composite Kompetitive Allele-Specific PCR Markers

**FIGURE 4:**
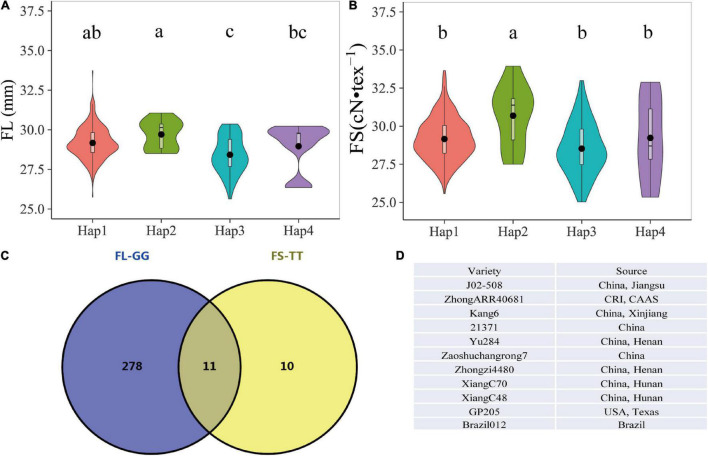
The effects of four haplotypes on fiber length and fiber strength. **(A)** Average phenotypic data of fiber length in multiple environments. **(B)** Average phenotypic data of fiber strength in multiple environments. **(C)** Eleven elite accessions with Hap2 genotypes. **(D)** List of 11 elite accessions with Hap2. Different lower case letters above the plots represent Duncan’s multiple comparisons at *P* < 0.05.

As both FL and FS were the important parameters in the manufacture of finer and stronger yarns, the pyramiding of genes, which involved the combining of two or more independent genes in a particular line, offered an efficient means to select elite materials for modern breeding (Sharma et al., 2004; Hu et al., 2012). Based on our previous study, the SNP D11_24030087 (G/T) for FL was also significantly associated with the FS trait (Ma et al., 2018). The combination of the above two KASP FMs resulted in four haplotypes (GC, GT, TC, and TT). Hap1 (GC) included 273 accessions with an average FL of 29.17 mm and FS 29.16 of cN•tex^–1^, Hap2 (GT) included 11 accessions with an average FL of 29.71 mm and FS of 30.69 cN•tex^–1^, Hap3 (TC) included 55 accessions with an average FL of 28.42 mm and FS of 28.53 cN•tex^–1^, and Hap4 (TT) included 9 accessions with an average FL of 28.96 mm and FS of 29.23 cN•tex^–1^. One-way ANOVA indicated that the haplotypes based on these two markers had significant effects on FL and FS. The accessions with Hap2 showed the highest averaged values in FL and FS. The multiple comparison analyses showed that Hap2 genotypes had significant differences from Hap3 and Hap4, while FS genotypes exhibited significant differences from Hap1. Therefore, Hap2 with two elite alleles was considered as the most favorable haplotype (Figures 4A,B and Table 4). The reliability of the haplotypes was also validated using different phenotypic data under each environment, and the results displayed were largely consistent with those observed *via* average data (Supplementary Tables 4, 5). Moreover, it was observed that the appeared frequency of accessions with Hap2 and FL longer than 28.5 mm (the mean of Hap2) was 100%, while the frequency of varieties with only the single elite allele G of the Hap2 and FL longer than 28.5 mm was 77.9%. Similar results were observed for FS, the frequency of varieties with Hap2 and FS higher than 29.06 cN•tex^–1^ was 72.7%, whereas the frequency of varieties with only the single elite allele T of the Hap2 and FS higher than 29.06 cN•tex^–1^ was 61.9% (Tables 3, 4). Obviously, Hap2 had an advantage over a single marker for MAS. Hence, Hap2 is the first recommended haplotype for selecting materials of higher FL and FS. Furthermore, 11 superior accessions harboring Hap2 were identified (Figures 4C,D), which provided superior materials for fiber quality improvement in the future breeding of upland cotton.

**TABLE 4:** The effects and frequencies of different haplotypes on FL and FS in the population of 360 accessions.

Haplotypes	$\bar{\text{x}}$ ± sd	Frequency * (%)	*P*	
	FL (mm) FS (cN•tex^–1^)	FL ≥ 28.50 FS ≥ 29.06	FL	FS
Hap1(273)	29.17 ± 1.00 29.16 ± 1.40	211 (77.3) 138 (50.5)	5.11E-06	0.0002
Hap2(11)	29.71 ± 0.90 30.69 ± 2.00	11 (100.0) 8 (72.7)		
Hap3(55)	28.42 ± 1.15 28.53 ± 1.73	24 (43.6) 19 (34.5)		
Hap4(9)	28.96 ± 1.49 29.23 ± 2.77	7 (77.8) 4 (44.4)		

$\bar{x}$, average phenotypic data under eight environments; SD, standard deviation; *, the percentage of accessions with the elite allele and higher in FL and FS than the mean of accessions with non-elite alleles.

### Further Validation of Haplotype Application

To further validate the efficacy of the haplotype in an application, we analyzed the cotton core collection with known phenotype and genotype based on our previous study (Ma et al., 2018). The results displayed that the FL and FS of varieties harboring different haplotypes were significantly different, e.g., the varieties harboring Hap2 with higher FL and FS of 30.65 mm and 28.83 cN•tex^–1^, respectively, than those with other haplotypes. In addition, the frequency of varieties harboring Hap2 and with FL longer than 28.0 mm (> the mean of accessions with non-elite allele) or FS higher than 26.90 cN•tex^–1^ (> the mean of accessions with non-elite allele) was also more often observed (100.0 and 96.7%) than that of varieties harboring other haplotypes (Table 5). These results consolidated the practicality and reliability of the Hap2 for the selection of superior varieties.

**TABLE 5:** The effects and frequencies of different haplotypes in the core cotton collection of 419 accessions.

Haplotypes	$\bar{\text{x}}$ ± sd	Frequency* (%)	*P*	
	FL (mm) FS (cN•tex^–1^)	FL ≥ 28.00 FS ≥ 26.90	FL	FS
Hap1(248)	29.52 ± 1.10 27.14 ± 1.54	230 (92.7) 154 (62.1)	3.50E-28	1.30E-18
Hap2(30)	30.65 ± 1.20 28.83 ± 1.32	30 (100.0) 29 (96.7)		
Hap3(79)	27.74 ± 1.71 26.37 ± 1.22	43 (54.4) 27 (34.2)		
Hap4(10)	29.26 ± 1.60 28.16 ± 2.06	8 (88.9) 8 (88.9)		

$\bar{x}$, average phenotypic data under 12 environments; SD, standard deviation; *, the percentage of accessions with the elite allele and higher in FL and FS than the mean of accessions with non-elite alleles; 419 lines from Ma et al. (2018).

## Discussion

Cotton fiber is the primary raw material in the textile industry (Kohel et al., 2001). With the advancements in techniques and diversified methods of spinning, greater fiber quality for processing is required. FL and FS are inherited quantitative traits (Paterson et al., 2003). A large number of genetic markers that contribute to FL and FS traits were identified *via* genome-wide association studies (Fang et al., 2017; Wang et al., 2017; Liu et al., 2018; Ma et al., 2018; Su et al., 2018; Zhang et al., 2019). However, the use of molecular markers is still limited in crop breeding at present. One major reason for this is the lack of effective markers with the high selective value of the interesting traits for breeders. The FMs developed from functional gene sequences can accurately discriminate alleles at a single locus and represent ideal markers for MAS in breeding, because they can fully diagnose the target trait allele. Additionally, the FMs in the genes allow selection in different genetic backgrounds without revalidating (Varshney et al., 2005).

In this study, two KASP FMs were designed from specific characteristics of *GhFL2* and *Gh_A07G1769*. The *GhFL2* was mapped on the fiber-related region from 24.02 to 24.09 Mb in Dt11 where one SNP D11_24030087 (G/T) was located on the coding sequences of *GhFL2*, resulting in the change of amino acid, and showed two different genotypes corresponding to differential FL (Ma et al., 2018). Moreover, this gene was also detected by another study with a non-synonymous mutation in the first exon (G to T transition) (Li et al., 2020). Another gene *Gh_A07G1769* was mapped from 72.17 to 72.23 Mb in At07. Three SNPs, namely, A07_72203768, A07_72204322, and A07_72204443, were located on the coding sequence and formed two haplotypes. The varieties with different genotypes showed significant differences in FS. Therefore, these variant loci were considered promising hubs to develop FMs. The genotyping results showed that two markers (D11_24030087 (G/T) and A07_72204443) could clearly distinguish the accessions and showed a high consistency between the genotyping results and phenotypic evaluation (Figures 2, 3 and Table 3). Statistical analyses with *t*-tests showed a significant difference between clusters, which is consistent with the previous studies (Ma et al., 2018; Li et al., 2020). These findings suggest that the FMs can be effective in more extensive populations to differentiate the elite genes in different varieties and will be useful for MAS in cotton breeding programs.

Additionally, we evaluated the potential application value of the FMs. We demonstrated that the varieties harbored G-allele (D11_24030087 G/T) with an average FL of 29.19 mm was longer than those harbored with T-allele with an average of 28.50 mm, indicating the elite allele “G” for increasing FL. Similarly, the varieties that possessed “T” from A07_72204443 C/T with an average FS of 30.15 were higher than those that possessed “C” with an average of 29.06 cN•tex^–1^. Therefore, we defined that the varieties harboring “G” and with FL longer than 28.5 mm and the varieties harboring “T” with FS higher than 29.06 cN•tex^–1^ were superior materials in the fiber properties, respectively. The investigation of the frequencies of varieties with elite alleles “G” or “T” indicated that the varieties with FL longer than 28.50 mm or FS higher than 29.06 cN•tex^–1^ accounted for 77.9 and 61.9%, respectively (Table 3), suggesting that these elite alleles existed in the most superior varieties and could be valuable for MAS. However, SNP combinations should be more persuasive than the single markers (Akey et al., 2001; Daly et al., 2001; Zill et al., 2004). In contrast, the pyramid of multiple target traits was preferred by breeders (Sharma et al., 2004; Pham et al., 2010, 2012; Salameh et al., 2011; Hickey et al., 2017). Toward these, we obtained more conclusive results from the haplotype analyses. The two KASP markers formed four haplotypes, and the appeared frequencies of varieties harbored Hap2 and FL longer than 28.5 mm and FS higher than 29.06 cN•tex^–1^ were 100 or 72.7%, respectively, which increased by 22.1 or 10.8% compared with that of single tag (77.9 and 61.9%, respectively) (Tables 3, 4). In addition, although no significant difference was observed between Hap1 and Hap2 based on the analyses of FL, Hap1 contained only “G” elite allele and was not the better choice for MAS; a significant difference was observed between Hap2 and other haplotypes based on the analyses of FS. Thus, Hap2 was considered as favorable haplotypes for selecting materials of superior FL and FS. The availability of the haplotype further validation in the cotton core collection delivered similar results to those mentioned earlier. Therefore, we recommend that Hap2 was the best haplotype to increase FL and FS. These FMs can be used in the KASP platform to query a large germplasm collection to identify favorable alleles at the target loci and can serve as a diagnostic tool for MAS in breeding programs for increased efficiency and precision at any stage of plant growth. Notably, FL and FS traits are quantitative characters in cotton, and they are controlled by multiple genes and have complex genetic behaviors (Paterson et al., 2003; Wilkins and Arpat, 2005; Said et al., 2013), and therefore, these markers may not the only criteria for selection.

## Conclusion

In this study, two functional SNP markers in two genes, namely, D11_24030087 and A07_72204443, were successfully developed using a rapid and cost-effective method available to date, the KASP assay. These two markers could effectively discriminate target genotypes that provided an improved accuracy. The Hap2 (GT) based on the two FMs to assist selection could be more efficient in the breeding of upland cotton.

## Data Availability Statement

The raw data supporting the conclusions of this article will be made available by the authors and available from the corresponding author on reasonable request.

## Author Contributions

ZM designed the study and revised the manuscript. LL completed genotyping the materials and wrote the manuscript. ZS, YZ, HK, JY, ZL, LW, and GZ performed phenotyping experiments. LL and XW analyzed data. All authors read and approved the manuscript.

## Conflict of Interest

The authors declare that the research was conducted in the absence of any commercial or financial relationships that could be construed as a potential conflict of interest.

## Publisher’s Note

All claims expressed in this article are solely those of the authors and do not necessarily represent those of their affiliated organizations, or those of the publisher, the editors and the reviewers. Any product that may be evaluated in this article, or claim that may be made by its manufacturer, is not guaranteed or endorsed by the publisher.
