# Supplementary material for: Development and Utilization of Functional Kompetitive Allele-Specific PCR Markers for Key Genes Underpinning Fiber Length and Strength in Gossypium hirsutum L
Source: Front Plant Sci. 2022 Mar 14;13:853827. doi: 10.3389/fpls.2022.853827 (PMC8964280; doi:10.3389/fpls.2022.853827)
Supplement: Supplementary file 1 [file Data_Sheet_1.docx]

**Supplement Table S2** Allelic effects of gene underpinning FL in the population under eight environments.

| Environments | Phenotype |  | G-AVE  (mm) | T-AVE  (mm) | Allele effect | *P*-value |
| --- | --- | --- | --- | --- | --- | --- |
|  | GG TT |  |  |  |  |  |
| 14-BD | 289 64 |  | 29.37 | 28.37 | 1.00 | 3.45E-05 |
| 14-HJ | 289 64 |  | 28.87 | 28.36 | 0.51 | 0.0091 |
| 14-XJ | 289 64 |  | 30.25 | 29.62 | 0.62 | 0.0020 |
| 14-QX | 289 64 |  | 29.66 | 28.80 | 0.86 | 2.55E-05 |
| 14-HN | 289 64 |  | 29.32 | 28.79 | 0.53 | 0.00169 |
| 15-XJ | 289 64 |  | 30.17 | 29.49 | 0.68 | 0.0003527 |
| 15-QX | 289 64 |  | 26.67 | 26.33 | 0.34 | 0.072 |
| 15-HN | 289 64 |  | 29.20 | 28.23 | 0.97 | 6.43E-07 |

**Supplement Table S3** Allelic effects of gene underpinning FS in the population under eight environments.

| Environments | Phenotype |  | C-AVE  (cN•tex^−1^) | T-AVE  (cN•tex^−1^) | Allele effect | *P*-value |
| --- | --- | --- | --- | --- | --- | --- |
|  | CC TT |  |  |  |  |  |
| 14-BD | 331 21 |  | 29.29 | 30.95 | 1.66 | 0.00353 |
| 14-HJ | 331 21 |  | 29.09 | 30.35 | 1.26 | 0.0125 |
| 14-XJ | 331 21 |  | 30.73 | 32.28 | 1.55 | 0.00168 |
| 14-QX | 331 21 |  | 30.00 | 31.37 | 1.37 | 0.0000 |
| 14-HN | 331 21 |  | 28.37 | 29.87 | 1.50 | 0.00175 |
| 15-XJ | 331 21 |  | 30.99 | 31.12 | 0.13 | NA |
| 15-QX | 331 21 |  | 28.36 | 28.88 | 0.52 | NA |
| 15-HN | 331 21 |  | 25.58 | 26.42 | 0.84 | 0.0367 |

**Supplement Table S4** Effects of different haplotypes on fiber length in the population under multi-environments.

| Environments | Haplotypes | | | | *P-value* |
| --- | --- | --- | --- | --- | --- |
|  | Hap1 | Hap2 | Hap3 | Hap4 |  |
| 14-BD | 29.33±1.66 | 29.95±2.27 | 28.19±1.73 | 29.46±2.66 | 0.000 |
| 14-HJ | 28.85±1.41 | 29.36±1.13 | 28.34±1.50 | 28.41±1.60 | 0.043 |
| 14-XJ | 30.25±1.41 | 30.30±1.00 | 29.47±1.55 | 30.57±1.98 | 0.002 |
| 14-QX | 29.63±1.43 | 30.47±1.33 | 28.71±1.51 | 29.30±1.91 | 0.000 |
| 14-HN | 29.29±1.19 | 29.94±1.31 | 28.71±1.28 | 29.23±1.44 | 0.003 |
| 15-XJ | 30.18±1.33 | 30.31±0.82 | 29.45±1.52 | 29.73±1.39 | 0.003 |
| 15-QX | 26.62±1.35 | 27.05±1.21 | 26.28±1.41 | 26.66±1.40 | 0.199 |
| 15-HN | 29.16±1.33 | 30.15±1.21 | 28.21±1.71 | 28.32±1.02 | 0.000 |

**Supplement Table S5.** Effects of different haplotypes on fiber strength in the population under multi-environments.

| Environments | Haplotypes | | | | *P-value* |
| --- | --- | --- | --- | --- | --- |
|  | Hap1 | Hap2 | Hap3 | Hap4 |  |
| 14-BD | 29.31±2.35 | 31.33±3.98 | 28.59±2.47 | 30.15±4.51 | 0.005 |
| 14-HJ | 29.16±2.15 | 30.99±2.21 | 28.69±2.31 | 29.24±3.50 | 0.021 |
| 14-XJ | 30.79±2.07 | 32.17±2.20 | 30.34±2.51 | 30.02±2.95 | 0.024 |
| 14-QX | 30.16±2.04 | 32.21±2.28 | 29.21±2.11 | 29.85±3.28 | 0.000 |
| 14-HN | 30.16±2.04 | 32.21±2.28 | 29.21±2.11 | 29.85±3.28 | 0.000 |
| 15-XJ | 30.18±1.33 | 30.31±0.82 | 29.45±1.52 | 29.73±1.39 | NA |
| 15-QX | 28.51±2.23 | 28.77±2.95 | 27.45±2.61 | 28.56±2.63 | 0.02 |
| 15-HN | 25.70±1.74 | 27.50±2.03 | 24.95±1.77 | 25.02±1.46 | 0.0002233 |
